# Probiotic *Bifidobacterium lactis* V9 attenuates hepatic steatosis and inflammation in rats with non-alcoholic fatty liver disease

**DOI:** 10.1186/s13568-020-01038-y

**Published:** 2020-05-29

**Authors:** Yan Yan, Chunyan Liu, Shimin Zhao, Xinxu Wang, Jinling Wang, Heping Zhang, Yuzhen Wang, Guofen Zhao

**Affiliations:** 1 College of Life Science, Inner Mongolia Agricultural University, Hohhot, 010018 People’s Republic of China; 2 College of Veterinary Medicine, Inner Mongolia Agricultural University, Hohhot, 010018 People’s Republic of China; 3 Key Laboratory of Dairy Biotechnology and Bioengineering, Educational Ministry of China, College of Food Science and Engineering, Inner Mongolia Agricultural University, Hohhot, 010018 People’s Republic of China

**Keywords:** Probiotics, NAFLD, AMPK, TLR, NF-κB

## Abstract

Both steatosis and inflammation are key pathological events in the progression of non-alcoholic fatty liver disease (NAFLD). Probiotics are beneficial for the prevention and treatment of NAFLD. *Bifidobacterium animalis* subsp*. lactis* V9 (V9) is a newly isolated strain with favorable probiotic properties. The study aims to evaluate the effects and mechanisms of V9 on the hepatic steatosis and inflammatory responses in a rat model of NAFLD induced by high-fat diets (HFD). Our results showed that administration of V9 significantly attenuated the HFD-induced increases in alanine transaminase (ALT) and aspartate aminotransferase (AST) levels, resulting in alleviated hepatic steatosis. V9 supplementation reduced the accumulation of hepatic triglyceride and free fatty acid,while increasing the levels of glycogen. Serum levels of glucose were also decreased in HFD rats administrated with V9. Meanwhile, the transcription of SREBP-1c and FAS was reduced, and the hepatic expression of phosphorylated-AMPK and PPAR-α was restored after V9 administration. V9 suppressed the production of inflammatory cytokines (e.g. IL-6, IL-1β, and TNF-α) in HFD-fed rats. The anti-inflammatory effects of V9 was found to be associated with the inhibition of hepatic expression of TLR4, TLR9, NLRP3, and ASC mRNA. Furthermore, the activation of ERK, JNK, AKT and NF-κB were suppressed by V9 treatment. These results indicate that *Bifidobacterium lactis* V9 improves NAFLD by regulating de novo lipid synthesis and suppressing inflammation through AMPK and TLR-NF-κB pathways, respectively.

## Key points

V9 supplementation alleviates HFD-induced metabolic disorder.

V9 inhibits HFD-induced expression of FAS, SREBP1c and activates AMPK.

V9 suppresses the TLR-NF-κB signaling pathway in HFD-fed rats.

## Introduction

Non-alcoholic fatty liver disease (NAFLD) is the chronic liver pathology that occurs when fat is deposited (steatosis) in the liver in the absence of a history of excessive alcohol consumption. NAFLD may progress to nonalcoholic steatohepatitis (NASH) with the help of fat deposition, oxidative stress and chronic inflammation in the liver. Some NAFLD patients may advance to develop cirrhosis and eventually hepatocellular carcinoma (Hashimoto and Tokushige 2011). The prevalence of NAFLD has been rapidly increasing due to lifestyle changes, such as increased consumption of high-fat diets (HFD) and the lack of exercise (Williams et al. 2011). NAFLD is now recognized as a worldwide health concern and it is closely associated with diabetes mellitus (type II) and cardiovascular diseases (Ratziu et al. 2010; Chacko and Reinus 2016). Besides lifestyle modifications (Malaguarnera et al. 2012) and bariatric procedures (Shouhed et al. 2017), there is no effective pharmacological treatment for NAFLD.

The pathogenesis of NAFLD is largely unknown. The widely accepted “two-hit” theory holds that the “first-hit” comprises of dysregulated lipid metabolism and insulin resistance followed by the hepatic “second-hit” including inflammatory cytokine production, oxidative stress-induced lipotoxicity and other mechanisms (Musso et al. 2010). Growing evidence indicates that the gut microbiota participates in NAFLD pathogenesis and lipotoxicity (Safari and Gérard 2019; Borrelli et al. 2018), through the metabolism of nutrients and a number of secretory factors that eventually target the liver (Szabo et al. 2011; Lu et al. 2016). Both experimental and clinical studies have shown that probiotics are beneficial in the prevention and treatment of NAFLD (Maddur and Neuschwander-Tetri 2014; Nobili et al. 2018). Administration of probiotic mixture of VSL#3 reduces HFD-induced inflammatory response in ApoE ^−/−^ mice (Chan et al. 2016). However, the molecular mechanisms of the beneficial effects of probiotics are not entirely understood.

*Bifidobacterium animalis* subsp*. lactis* V9 (V9) is a novel strain isolated from healthy Mongolian children, possessing favorable probiotic properties such as aciduricity and bile resistance (Sun et al. 2010). The strain has been used in the industrial fermentation of dairy starter cultures by Inner Mongolia Yili Industrial Group Co. Ltd, China. The results of a recent research have demonstrated that the fermentation of whole oat flour with V9 and *Lactobacillus plantarum* TK9 yield a synbiotic food rich in lactic acid bacteria and prebiotics (Wang et al. 2015). A study has shown that *Bifidobacterium* and dietary blueberry supplementation could attenuate hepatic lipid accumulation in NAFLD rats (Ren et al. 2014). Moreover, administration of *Bifidobacterium longum* and fructo-oligosaccharides reduces the production of pro-inflammatory cytokines, steatosis and NASH in patients (Malaguarnera et al. 2012). We are wondering if the novel probiotic V9 strain has beneficial effects for NAFLD patients. In this study, we established an HFD-induced rat model of NAFLD that reflects the path physiology of the disease in humans, including the development of fatty liver, hyperglycemia, as well as systemic inflammation. We then explored the potential effects and underlying mechanisms of V9 on the improvement of metabolic dysfunction and inflammation in HFD-fed rats by examining various physiological parameters, the changes of metabolism-related genes and the inflammatory signaling pathways. Our results indicate that *Bifidobacterium lactis* V9 is a potential candidate for the treatment of HFD-induced metabolic syndrome.

## Materials and methods

### Bacteria strain and growth conditions

The strain of *Bifidobacterium animalis subsp. lactis* V9 (V9) is a special probiotic starter identified and preserved by the Key Laboratory of Dairy Biotechnology and Bioengineering, Educational Ministry of China, and also submitted to CGMCC for preservation with a deposit number of No. 5470. The bacteria was isolated from healthy Mongolian children and identified by PCR-based 16 s rRNA amplication. The V9 strain shares 99% homology with *Bifidobacterium animalis* subsp. *lactic* Bb12 (Sun et al. 2010). The strain was cultured in a modified TPY agar medium under an anaerobic environment at 37 °C. The V9 cell pellets were harvested, washed twice with PBS and then lyophilized. The lyophilized powder of V9 was resuspended in physiological saline and adjusted to 1 × 10^9^ CFU/ml for treatment of the rats.

### Animal experiments

Male Wistar rats (120–140 g) were purchased from Vital River Laboratories Animal Co. Ltd. (Beijing, China) and housed under controlled conditions (20–22 °C, 55 ± 10% relative humidity, 12-h light/dark cycles). Experiments on rats began after 1 week of adaptation. During the adaption period, rats were fed a standard chow diet (10% energy from fat, Ke Ao Xie Li Diet Co. Ltd, Beijing, China) and received water ad libitum. All protocols for animal experiments were approved by the Animal Care and Use Committee at Inner Mongolia Agricultural University. Rats were randomly divided into five groups (n = 8 in each group): control, high-fat diet (HFD), HFD + Berberine, V9 treatment (HFD/V9) and V9 control (CON/V9). With the exception of the control and CON/V9 group, which were fed with a normal chow diet, rats in the remaining groups were given a high-fat diet (60% energy from fat, Ke Ao Xie Li Diet Co. Ltd, Beijing, China) to establish a model of NAFLD. After 6 weeks, all groups of rats were fed with a normal chow diet. Rats in the HFD/V9 and CON/V9 groups were gavaged with V9 (1 × 10^9^ CFU) for 4 weeks. At the same time, the HFD + Berberine group of rats received oral administration of Berberine (300 mg/kg). At the end of the experiment, all the rats were fasted overnight and then anesthetized with isoflurane. Samples of blood and liver were collected. Parts of liver tissues were fixed in 10% formalin for histological analysis and other samples were stored in aseptic cryopreservation tubes. The tubes were immediately snap-frozen in liquid nitrogen followed by storage in a − 80 °C refrigerator.

### Biochemical analysis

Serum samples were obtained by centrifuging the clotted blood samples at 3500×*g* for 10 min, which was then stored at − 80  °C for subsequent analysis. The levels of serum ALT, AST and glucose were measured using an automatic biochemical analyzer (Olympus 2700). Hepatic triglyceride (TG), glycogen, and free fatty acid (FFA) levels were measured by the corresponding kits according to the manufacturer’s instructions (Jiancheng Institute of Biotechnology, Nanjing, China). The lysate of liver samples (100–200 mg) was prepared by homogenization in modified RIPA buffer with protease inhibitor cocktail. The samples were processed mechanically by a dounce homogenizer with 15–20 strokes. Tissue and cell debris in the lysates was then removed by centrifugation. The protein concentration was quantified using the Sangon Biotech BCA protein kit according to the manufacturer’s instructions. The contents of TG, glycogen, and FFA were measured by spectrophotography and calculated based on the protein content of the liver homogenates.

### Histological examination

The liver samples were fixed in 10% paraformaldehyde at room temperature for 24 h. After dehydration through a graded ethanol series, the samples were immersed in xylene, embedded in paraffin, and then cut into 5 μm sections for hematoxylin-eosin (H&E) staining. The images of HE-stained sections were taken using a light microscope and assessed blindly.

### Enzyme-linked immunosorbent assay

Serum TNF-α, IL-1β, and IL-6 levels were detected using commercially available enzyme-linked immunosorbent assay (ELISA) kits (R&D Systems, USA) according to the manufacturer’s instructions.

### Real-time PCR assay

Total mRNA from the liver tissues was extracted using a Trizol reagent (Invitrogen, Carlsbad, CA). RNA concentration and purity (OD260/280) were measured by a NanoDrop Lite spectrophotometer. The total mRNA (1 μg) was reversed into first strand cDNA using the RT-PCR kit (Takara, Dalian, China). Real-time PCR assay was performed using the specific primers as listed in Additional file 1: Table S1. GAPDH was used as an endogenous control to normalize the relative expression of target transcripts. Relative quantification of gene expression was performed by the 2^−ΔΔCt^ method.

### Western blot analysis

The liver proteins were extracted by using the tissue protein extraction solution or the nucleoprotein extraction kit (Sangon Biotech, Shanghai, China). The protein content was measured as previously described. The same amount of total protein from each sample was electrophoresed into SDS-PAGE gels and transferred to polyvinylidene difluoride (PVDF) membranes. The PVDF membranes were probed using rabbit polyclonal antibodies (Cell Signaling) against p-ERK1/2 (Thr202/Tyr204) (#9101), ERK1/2 (#9102), p-JNK(Thr183/Tyr185)(#9251), JNK (#9252), p-AKT (Ser473) (#9271), AKT(#4691), p-NF-κB (Ser468) (#3039), NF-κB (#3034) and p-AMPK (Thr172) (#2535), AMPK (#2532) overnight at 4 °C. After washing five times with TBST, the membranes were incubated with Goat anti-Rabbit IgG H&L (IRDye^®^ 800CW) for 1 h. The images of specific proteins were detected and analyzed using the LI-COR Odyssey CLx.

### Statistical analysis

The statistical analysis was performed using the SPSS 17 software package. All the data were expressed as mean ± standard deviation (SD) and analyzed using the one-way analysis of variance (ANOVA) with Duncan’s multiple-range test. P values of < 0.05 were regarded as statistically significant.

## Results

### V9 treatment attenuates HFD- induced liver injury

**Fig. 1:**
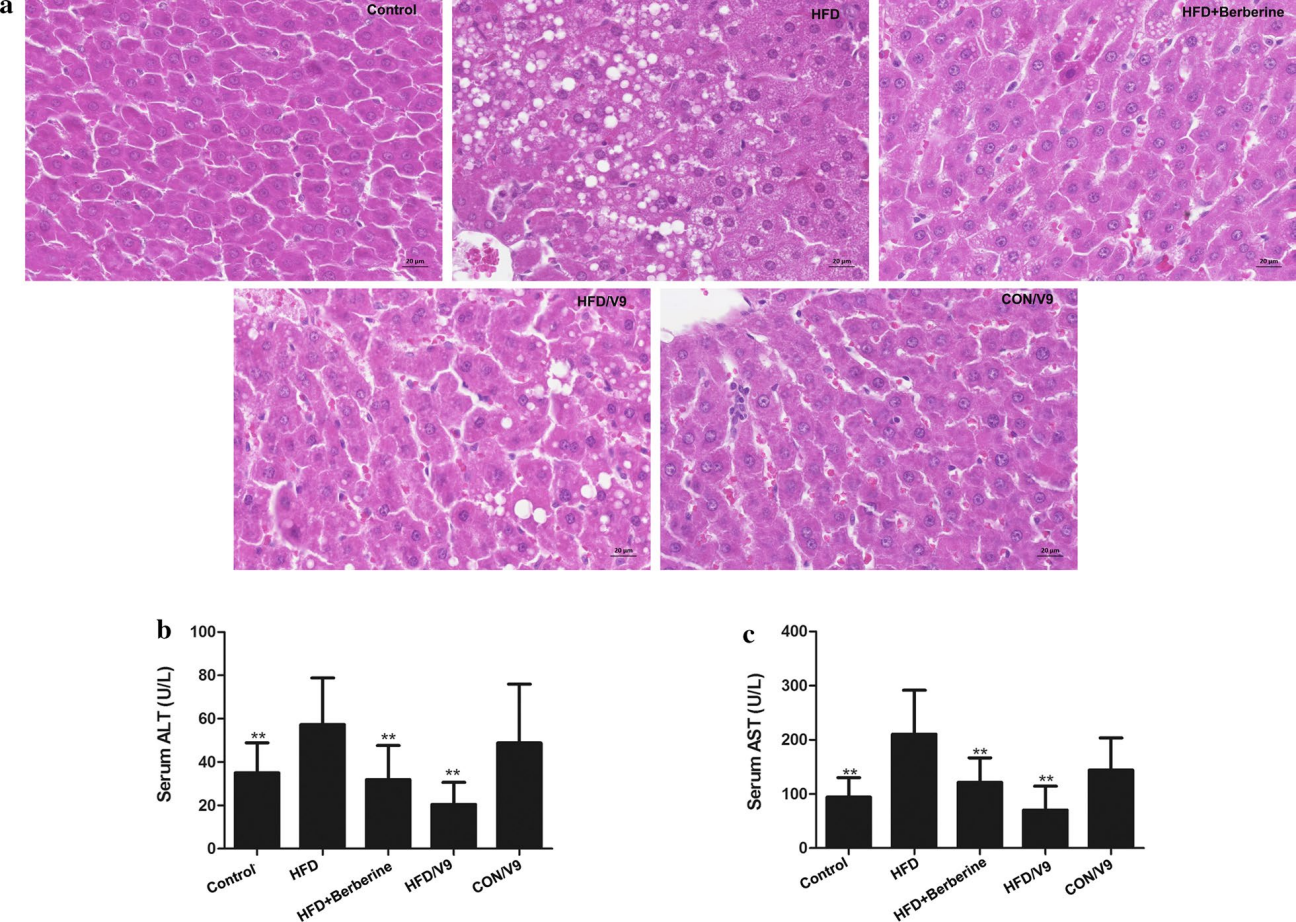
Probiotic V9 improves hepatic steatosis and liver damage in HFD rats. **a** Paraffin-embedded liver tissues (n = 3 each group) were sectioned and stained with hematoxylin and eosin(H&E). Micrographs are representative pictures with magnification 200×. Serum ALT (**b**) and AST levels were measured (n = 8, each group). **P < 0.01 vs HFD group

In order to explore the mitigating effects of *Bifidobacterium hactis* V9 on HFD-induced liver injury, the development of liver damage was examined histologically by H&E staining. Administration of the high-fat diet for 6 weeks caused evident damage in the liver as shown by the disturbed structure of lobules, hepatocyte hypertrophy and severe hepatic steatosis represented by the formation of numerous lipid droplets. In contrast, *Bifidobacterium lactis* V9 and Berberine treatment significantly improved the structure of liver lobules and alleviated hepatic steatosis (Fig. 1a). In addition, serum alanine transaminase (ALT) and aspartate aminotransferase (AST) levels were different across the various conditions. Compared with the HFD group, both V9 and Berberine treatment significantly decreased the serum ALT and AST levels (Fig. 1b and c). These results indicate that V9 treatment alleviates high fat diet-induced hepatic injury and steatosis.

### V9 treatment improves HFD-induced disorder in lipid and glucose metabolism

**Fig. 2:**
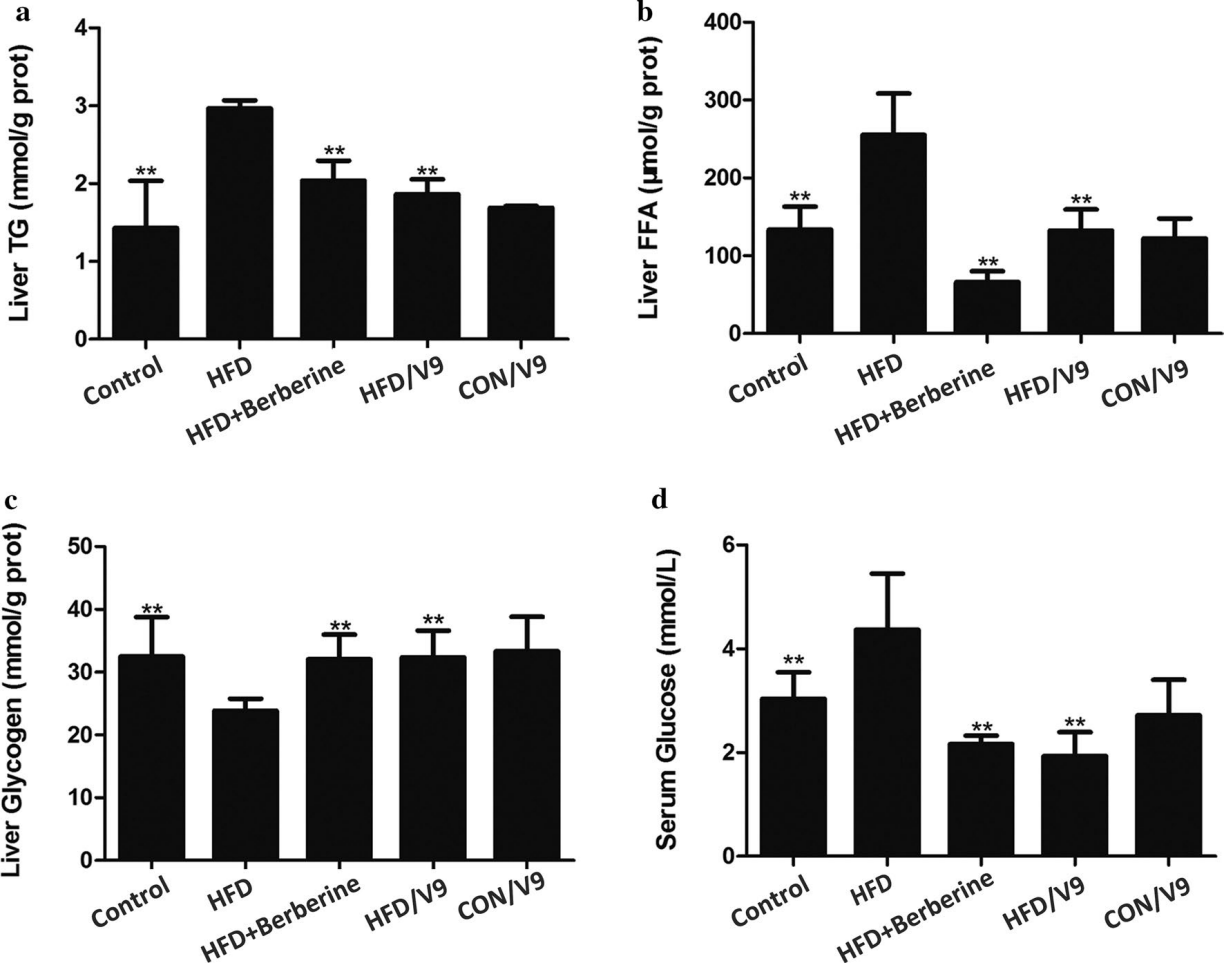
V9 treatment improves HFD-induced metabolic disorder. **a** Hepatic levels of TG. **b** Hepatic levels of FFA. **c** Hepatic levels of Glycogen. **d** Fasting glucose levels in serum. Data are expressed as mean ± SD with n = 8, **P < 0.01 vs HFD group

Next, we measured the parameters of lipid and glucose metabolism in the liver and the serum. The hepatic TG and FFA levels were increased in the HFD group, while these decreased in the V9 and Berberine treatment group (Fig. 2a and b). In addition, V9 and Berberine treatment restored the HFD-induced decrease in the hepatic glycogen content (Fig. 2c). Compared with the HFD group, V9 supplementation also induced a significant reduction in serum Total Cholesterol (TC) and fasting glucose levels (Fig. 2d). These results indicate that V9 supplementation improved the disorder in lipid and glucose metabolism.

### V9 supplementation reverses the deregulation of genes involved in lipid and glucose metabolism

**Fig. 3:**
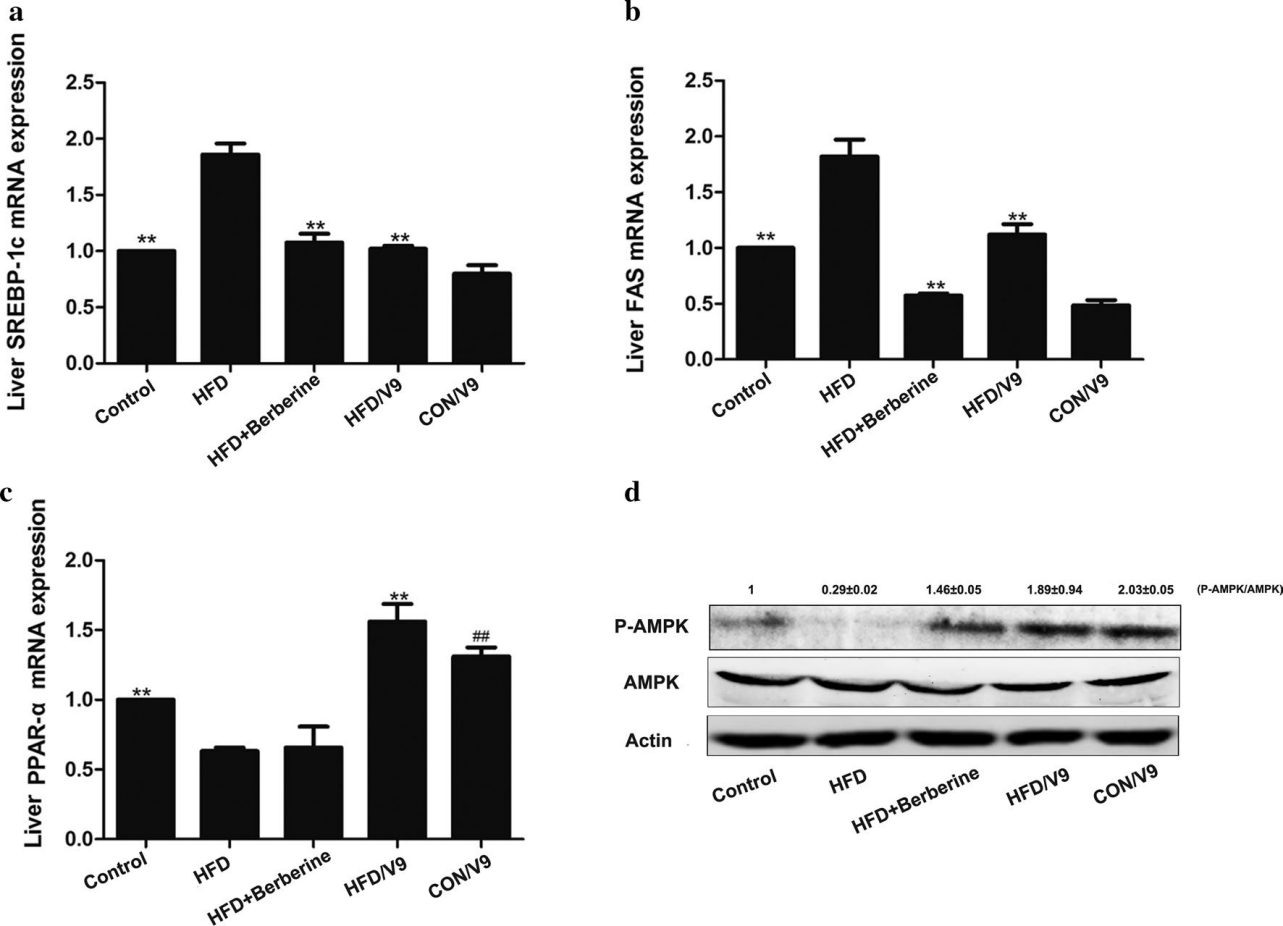
Probiotic V9 regulate the expression of the genes in lipid metabolism. Relative SREAP-1c (**a**), FAS (**b**), and PPAR-α mRNA expression (**c**) are reported (n = 8 each group). ^#^P < 0.05 vs control group, and **P < 0.01 vs HFD group. **d** Western blotting analysis of hepatic phospho-AMPK (p-AMPK) and total AMPK. The representative picture is one from three independent experiments. The densitometric analysis of bands from all samples was shown as the fold change relative to the control group

To understand the underlying mechanisms of the observed effects of V9 treatment, we evaluated the key signaling pathways involved in the modulation of lipid and glucose metabolism. The mRNA expression of sterol regulatory element-binding protein 1c (SREBP-1c) and the lipogenic enzyme fatty acid synthase (FAS) increased in HFD rats and was subsequently reduced by V9 and Berberine treatment (Fig. 3a and b). Hepatic PPAR-α mRNA expression was dramatically reduced by HFD challenge, which was restored by V9 treatment. On the other hand, there is no significant difference between the PPAR-α mRNA expression levels in the Berberine treatment group and the HFD group. Compared with the control group, an increase in the expression of PPAR-α mRNA was also observed in the CON/V9 group (Fig. 3c). Furthermore, western blotting analysis showed that treatment with V9 and Berberine restored the expression of phosphorylated-AMPK (Fig. 3d).

### V9 supplementation reduces HFD-induced pro-inflammatory responses

**Fig. 4:**
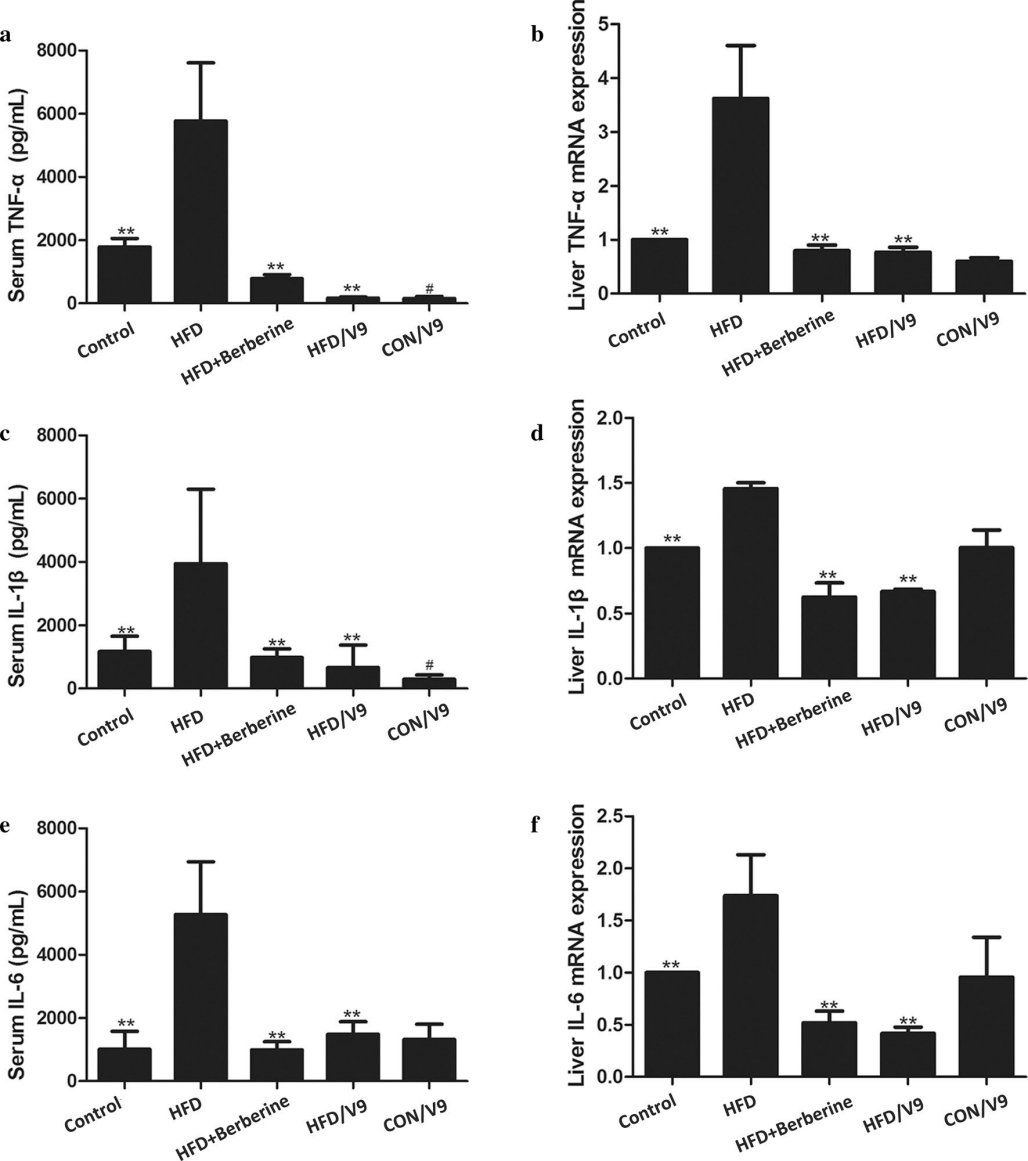
V9 treatment reduces HFD-induced inflammatory responses. **a** Serum TNF-α production. **b** Hepatic TNF-α mRNA expression. **c** Serum IL-1β production. **d** Hepatic IL-1β mRNA expression. **e** Serum IL-6 production. **f** Hepatic IL-6 mRNA expression. Data are expressed as mean ± SD, with n = 8, ^#^P < 0.05 vs control group, and **P < 0.01 vs HFD group

To investigate the anti-inflammatory effects of the probiotic V9, hepatic mRNA levels and serum concentrations of TNF-α, IL-1β, and IL-6 were measured. Compared with the control group, HFD challenge resulted in significantly increased levels of serum TNF-α, IL-1β and IL-6 which was also the case for the hepatic mRNA expression of these cytokines. V9 supplementation markedly inhibited HFD-induced expression of TNF-α, IL-1β, and IL-6 at both the mRNA and the protein level. A similar trend was found in the Berberine treatment group (Fig. 4a–f). These results indicate that both V9 and Berberine can reduce HFD-induced pro-inflammatory cytokine production.

### V9 suppresses the hepatic expression of TLRs and NLRP3

**Fig. 5:**
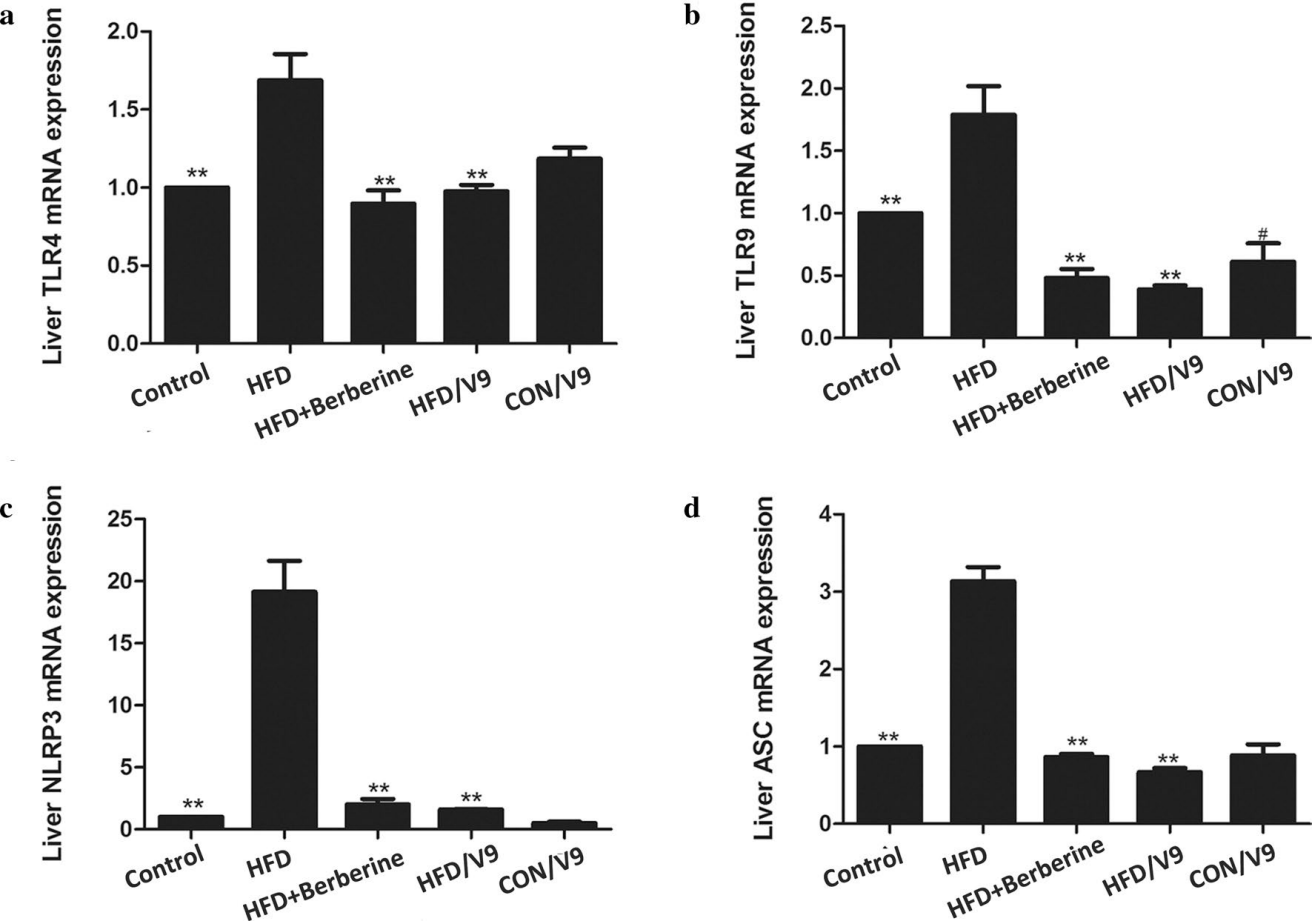
The effect of Probiotic V9 on hepatic TLRs, NLRP3 and ASC. **a** Hepatic mRNA levels of TLR4. **b** Hepatic mRNA levels of TLR9. **c** Hepatic mRNA levels of NLRP3. **d** Hepatic mRNA levels of ASC. Data are expressed as mean ± SD with n = 8, ^#^indicates P < 0.05 vs Control group; **P < 0.01 vs HFD group

As shown in Fig. 5a and b, the hepatic TLR4 and TLR9 mRNA expression increased significantly in the HFD group in comparison with the control group. However, V9 and Berberine treatment significantly reduced the expression of TLR4 and TLR9 mRNA in HFD rats. We found a similar pattern in the TLR4 and TLR9 protein expression after the administration of V9 or Berberine (Additional file 1: Figure S1). A significant decline in NLRP3 and ASC mRNA expression was also found in the V9 and Berberine treatment groups (Fig. 5c and d).

### V9 inhibits the activation of MAPK, AKT and NF-κB

**Fig. 6:**
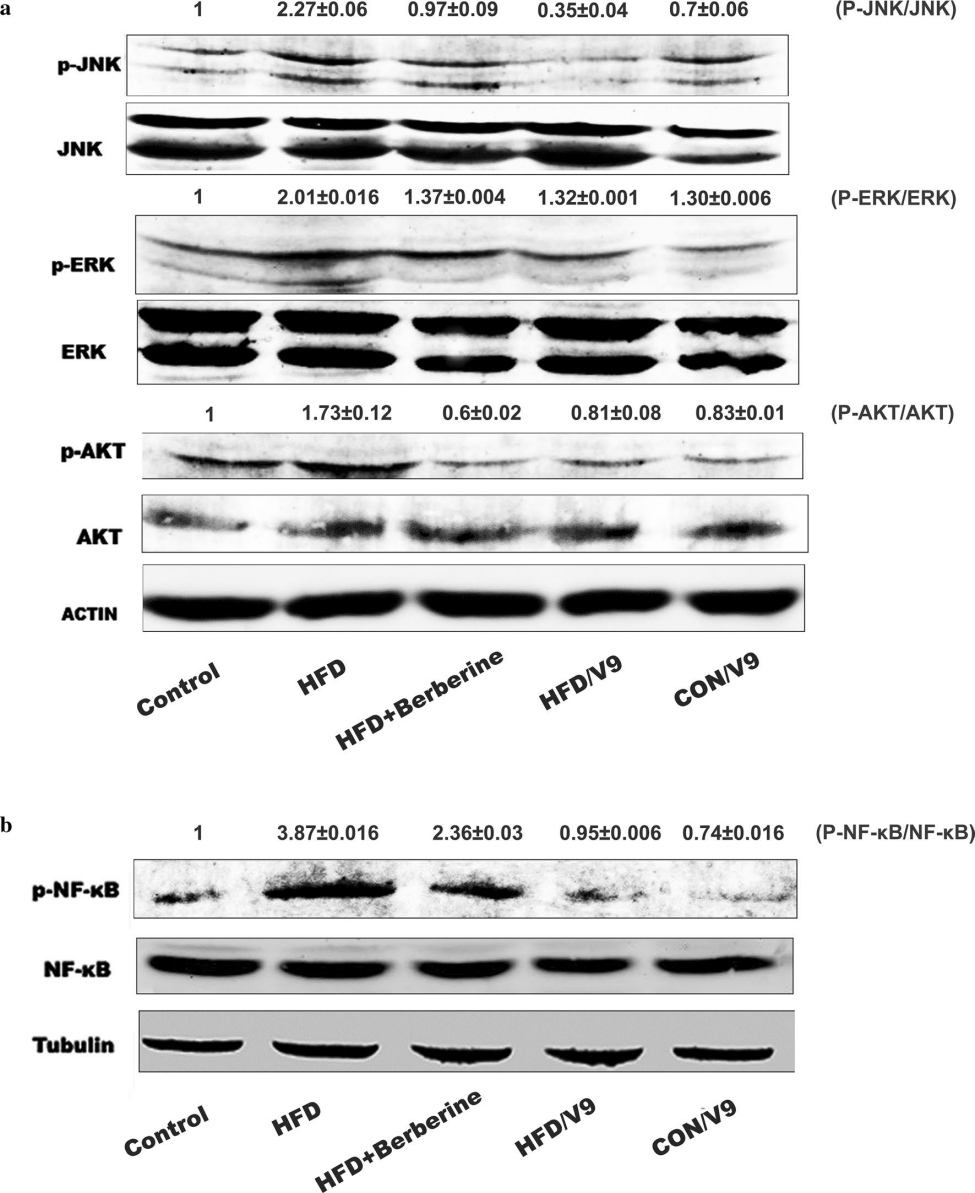
The effects of probiotic V9 on hepatic MAPK, AKT and NF-κB activation in HFD-challenged rats. **a** Western blotting analysis of phospho –JNK (p-JNK), total JNK, phospho-ERK (p-ERK), total ERK, phospho-AKT (p-AKT) and total AKT. **b** Western blotting analysis of nuclear phospho-NF-κB (p-NF-κB) and total NF-κB. The Representative pictures in **a** and **b** are shown from three independent experiments. The densitometric analysis of bands from all samples was shown as the fold change relative to the control group

In order to further study the mechanisms of the protective effects of V9 against HFD-induced NAFLD, we went on to evaluate the changes of the TLR-mediated downstream signaling pathways. As shown in Fig. 6a, HFD challenge resulted in elevated phosphorylation of JNK, ERK and AKT. However, V9 and Berberine supplementation evidently reduced the activation of JNK, ERK and AKT. V9 treatment also inhibited HFD-induced phosphorylation of NF-κB (Fig. 6b). These results indicate that modulation of the activation of MAPK, AKT and NF-κB contribute to the anti-inflammatory effects of V9.

## Discussion

The chronic liver disease NAFLD has emerged as a worldwide health burden, without effective treatments. Probiotics confer health benefits on the host with NAFLD by regulating glycolipid metabolism and steatosis-induced inflammatory responses (Kim et al. 2017; Ritze et al. 2014), which are dependent on the strains and the dosages (Xu et al. 2012; Ferolla et al. 2015). In this study, our results reveal that the novel strain of probiotic V9 exhibits beneficial effects in the HFD-induced NAFLD setting through its hypolipidemic and anti-inflammatory potential.

In our study, a rat model of NAFLD was successfully established, as evidenced by elevated serum glucose levels, compounded by the accumulation of hepatic TG and FFA. In addition, histopathological observations revealed severe steatosis in the NAFLD rat model. Studies have demonstrated that the levels of ALT and AST are increased in patients of NAFLD with insulin resistance (Sheng et al. 2018). Similarly, our study also shows that ALT and AST levels are significantly increased in HFD-challenged rats. All these results indicate that the HFD-challenged rats exhibit metabolic dysfunction, hepatic steatosis, and hepatotoxicity, which are in concordance with signs presented by NAFLD patients.

Evidence from a randomized, double-blind, placebo-controlled study demonstrated that administration of a multispecies probiotic mixture reduces the intrahepatic fat and body weight in obese NAFLD patients (Ahn et al. 2019). Animal studies have also shown that co-administration with cholesterol-lowering probiotics (two strains) and anthraquinone from *Cassia obtusifolia L.* has potential therapeutic effects for NAFLD (Mei et al. 2015). A similar study also indicates that supplementation with *Bifidobacterium longum* and *Lactobacillus acidophilus* attenuate liver fat accumulation in NAFLD rats, with a superior observed from treatment with *Bifidobacterium longum* (Xu et al. 2012). Our results are consistent with previously published studies, showing that V9 treatment improved hepatic steatosis and HFD-induced lipid and glucose disorder. Serum levels of ALT, AST, TG and glucose were markedly reduced, accompanied by reduced hepatic lipid accumulation (TG and FFA) and increased hepatic glycogen content in the V9 treatment group. These results suggest the single *Bifidobacterium* strain V9 has beneficial effects,and attenuates the progression of NAFLD. Whether the V9 strain is superior in terms its ability to alleviate liver fat accumulation than other identified *Lactobacillus* strains needs to be clarified in the future.

In order to elucidate the underlying mechanisms of the hypolipidemic effect of V9, the expression of lipogenic sterol regulatory element binding protein 1c (SREBP1c) and its target gene fatty acid synthase (FAS) was analyzed. The decreased expression of SREBP1c and FAS in the HFD/V9 group indicates that de novo lipid synthesis was suppressed by V9 treatment. Peroxisome proliferative-activated receptors (PPARs) are transcription factors belonging to the nuclear receptor superfamily, which are involved in lipid metabolism, energy homeostasis and inflammation (Derosa et al. 2017). PPARα is highly expressed in the liver, and is the master regulator of hepatic lipid flux by modulating fatty acid transport and β-oxidation. In addition, PPARα activation counteracts the expression of the inflammatory genes induced by NF-κB (Tanaka et al. 2017; Alves et al. 2017; Staels et al. 1998). Experimental evidence shows that PPARα deficiency leads to susceptibility to NAFLD, NASH and hepatic inflammatory responses (Ip et al. 2003). Thus, PPARα is an potential therapeutic target for hyperlipidemia. Research has shown that selective activation of PPARa by compound K-877 improves lipid metabolism in mice (Takei et al. 2017). The results of our study showed that HFD-induced decreased expression of hepatic PPAR-α mRNA was restored in the V9 treatment group but not in the Berberine group, suggesting different underlying mechanisms of action of V9 and Berberine.

AMP-activated kinase (AMPK), a key regulator in energy metabolic homeostasis, has been identified as a crucial target for drugs (Hardie et al. 2012). Activated AMPK phosphorylates SREBP and attenuates hepatic steatosis and atherosclerosis in mice with insulin resistance (Li et al. 2011). Treatment with V9 and Berberine induced the phosphorylation of AMPK, indicating that both of them alleviated hepatic steatosis through promoting the activation of AMPK. A recent study demonstrated that *Lactobacillus plantarum* NA136 could activate the AMPK signaling pathway to suppress the SREBP-1/FAS signaling, which in turn inhibits de novo lipogenesis in mice with NAFLD (Zhao et al. 2019). Our study is consistent with the study of *Lactobacillus plantarum* NA136. And thus, we conclude that V9 supplementation alleviates HFD-induced metabolic disorder through upregulating the AMPK and PPARα signaling pathways.

It has been demonstrated that TLR4 plays a key role in the progression from hepatic steatosis to NASH in mice with obesity-induced NAFLD (Ye et al. 2012). In addition, upregulation of TLR9 is increased in NASH models and activation of TLR9 signaling lead to inflammatory cell recruitment (Mridha et al. 2017a, b). Our results concur with previous reports by showing that the expression of TLR4 and TLR9 was increased in the NAFLD model group. However, V9 supplementation downregulated the expression of TLR4 and TLR9 (Fig. 5), which correlated with reduced production of pro-inflammatory cytokines in V9 treatment group. This suggests that the beneficial effects of V9 on NAFLD are associated with the downregulation of TLR4 and TLR9-induced inflammatory responses.

The role of NLRP3 in the progression from steatosis to NASH has also been documented recently (Rossato et al. 2020). Blocking NLRP3 by a small-molecule inhibitor MCC950 mitigates hepatic inflammation and fibrosis, as shown in two mice models of NASH (Mridha et al. 2017a, b). In addition, the study of Xiao et al. (2016) showed that bee honey attenuates the progression of NASH partly through suppressing the thioredoxin-interacting protein (TXNIP)-NLRP3 pathway. In our study, increased transcription of NLRP3 and its adaptor protein ASC was present in the NAFLD model group. Treatment with V9 and Berberine significantly decreased the expression of NLRP3 and ASC, suggesting that regulation on the expression of NLRP3 inflammasome is one of the underlying mechanisms of the anti-inflammatory effect of V9.

To further support the anti-inflammatory effect of V9 on HFD-induced inflammatory responses, we studied the TLR4 downstream signaling changes. Our results showed that V9 and Berberine treatment reduced the phosphorylation of JNK, ERK, AKT, and NF-κB (Fig. 6). As activation of NF-κB leads to the priming of NLRP3 inflammasome, it is rational to deduce that reduced activation of NF-κB contributes to the reduced expression of NLRP3, which was verified in our study. In summary, the anti-inflammatory effect of V9 against NAFLD is associated with the down-regulation on TLR-NF-κB-NLRP3 signaling.

In summary, our results demonstrate that the administration of probiotic V9 alleviates HFD-induced liver damage, mediated by a reduction in hepatic fat accumulation (through increasing AMPK-mediated fatty acid β-oxidation and reduced de novo lipid synthesis) and its anti-inflammatory activity (through downregulation of TLR-NF-κB signaling pathways). Therefore, we demonstrate that the probiotic V9 has the potential to be used as an treatment for NAFLD.

## Supplementary information

**Additional file 1: Table S1.** The specific primer sequences of genes of interest. **Figure S1.** Western blot analysis of hepatic TLR4 and TLR9. The representative picture is one from three independent experiments.

## Abbreviations

NAFLD: Non-alcoholic fatty liver disease
V9: *Bifidobacterium animalis* subsp*.lactis* V9
HFD: High-fat diets
ALT: Alanine transaminase
AST: Aspartate aminotransferase
TG: Triglyceride
FFA: Free fatty acid
NASH: Nonalcoholic steatohepatitis
CGMCC: China General Microbiological Culture Collection Center
HE: Hematoxylin–eosin
ELISA: Enzyme-linked immunosorbent assay
SREBP1c: Sterol regulatory element binding protein 1c
FAS: Fatty acid synthase
PPARs: Peroxisome proliferative-activated receptors
AMPK: AMP-activated kinase
