# Supplementary material for: Probiotic Bifidobacterium lactis V9 attenuates hepatic steatosis and inflammation in rats with non-alcoholic fatty liver disease
Source: AMB Express. 2020 May 29;10:101. doi: 10.1186/s13568-020-01038-y (PMC7260323; doi:10.1186/s13568-020-01038-y)
Supplement: Supplementary file 1 — Additional file 1: Table S1. The specific primer sequences of genes of interest. Figure S1. Western blot analysis of hepatic TLR4 and TLR9. The representative picture is one from three independent experiments. [file 13568_2020_1038_MOESM1_ESM.pdf]

Supplement material

Journal name : Applied Microbiology and Biotechnology

Title: Probiotic *Bifidobacterium Lactis* V9 attenuates hepatic steatosis and inflammation in rats with non-alcoholic fatty liver disease

Authors: Yan Yan, Chunyan Liu, Xinxu Wang, Xinyi Li, Jian Huang, Jinling Wang, Heping Zhang, Yuzhen Wang, Guofen Zhao

Corresponding authors:

Yuzhen Wang

E-mail: wangyuzhen817@126.com

Tel: +86-13848182817

Fax: +86-471-4309242

Guofen Zhao

E-mail: guofenzhao@126.com

Tel：+86-13948418594

Fax: +86-471-4309242

Table S1 The specific primer sequences of genes of interest

Gene	Primer Sequences
IL-6	Forward：5′-TAGTCCTTCCTACCCCAATTTCC-3′
	Reverse：5′-TTGGTCCTTAGCCACTCCTTC-3′
Il-1β	Forward：5′-TGGATGCTCTCATCAGGACAG -3′
	Reverse：5′-GAAATGCCACCTTTTGACAGTG -3′
TNF-a	Forward：5′-AGAACTCCAGCCGGTGTCTGTG-3′
	Reverse：5′-GTGGCAAATCGGCTGACGGTGT-3′
TLR4	Forward：5′- CACTCGAGGTAGGTGTTTCTGCTAA-3′
	Reverse：5′-GATTGCTCAGACATGGCAGTTTC -3′
TLR9	Forward：5′-TGATCACAGCGACGGCAATT -3′
	Reverse：5′-CCGAAGACCTAGCCAACCT -3′
SREBP-1c	Forward：5′-TGCCCTAAGGGTCAAAACCA-3′
	Reverse：5′-TGGCGGGCACTACTTAGGAA -3′
FAS	Forward：5′-AGGTTGGTGCACCTCCACTTG -3′
	Reverse：5′-GAGCGTTCGTGAAACCGACA-3′
PPAR-ɑ	Forward：5′-CTCTGATCCCTCTAGCACCTT-3′
	Reverse：5′-GGACAGCAAATCTTGAAGCAGC-3′
GAPDH	Forward：5’-TGTAGACCATGTAGTTGAGGTCA -3’
	Reverse：5′-AGGTCGGTGTGTGAACGGATTTG-3′

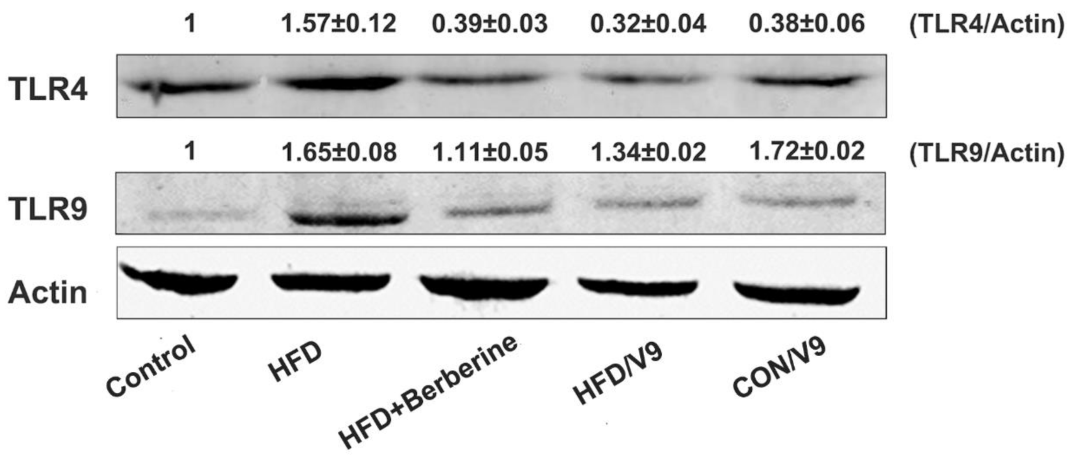

Figure S1 Western blot analysis of hepatic TLR4 and TLR9. The representative picture is one from three independent experiments.
